# Adult pulmonary tuberculosis as a pathological manifestation of hyperactive antimycobacterial immune response

**DOI:** 10.1186/s40169-016-0119-0

**Published:** 2016-09-21

**Authors:** Pawan Kumar

**Affiliations:** Special Centre for Molecular Medicine, Jawaharlal Nehru University, New Mehrauli Road, New Delhi, 110067 India

**Keywords:** Tuberculosis, Protective immunity, Pathogenesis, Interferon-gamma, Disease heterogeneity

## Abstract

The intricate relationship between tuberculosis (TB) and immune system remains poorly understood. It is generally believed that weakening of the immune response against *Mycobacterium tuberculosis* leads to reactivation of latent infection into the active pulmonary disease. However, heterogeneous nature of TB and failure of rationally designed vaccines in clinical trials raises serious questions against the simplistic view of TB as an outcome of weakened immunity. In the wake of accumulating human TB data, it is argued here that a hyperactive antimycobacterial immune response is to blame for the pathogenesis of pulmonary TB in immunocompetent adults. Direct and indirect evidence supporting this notion is presented in this article. Revisiting the role of immune system in TB pathogenesis will pave the way for effective anti-TB vaccines.

## Background

Tuberculosis (TB) continues to be one of major global health threats resulting in nearly 1.5 million deaths per year [1]. An effective vaccine is urgently required to control current specter of TB, but the development of new TB vaccines has been hampered due to poor understanding of the intricate relationship between TB and immune system. It is commonly believed that weakening of the immune system leads to reactivation of latent *Mycobacterium tuberculosis* (*Mtb*) infection into the active pulmonary disease. Although a major support to this notion is provided by the significantly elevated risk of TB in immunocompromised hosts (such as those suffering from HIV/AIDS), TB in immunocompromised people is different from that occurring in immunocompetent adults.

In immunocompetent adults, TB occurs as a post-primary and localized disease affecting mainly lung tissue. On the contrary, *Mtb* infection in immunocompromised hosts leads to a primary progressive disease, commonly affecting extrapulmonary sites and often occurring in a severe form. Owing to its heterogeneous presentation and pattern of occurrence, an increasing number of researchers have been questioning the simplistic view of TB as an outcome of weakened immune response. Failure of rationally designed vaccines, e.g. MVA85A (modified vaccinia virus Ankara expressing *Mtb* antigen 85A), which had been aimed at boosting antimycobacterial immune response, further raises the serious concerns about our current understanding of the TB immunobiology.

On the other hand, a number of experimental and observational studies have shown that a heightened immune response is closely associated with active pulmonary TB or its elevated risk in immunocompetent adults. Based on these and other findings, it is proposed here that a hyperactive antimycobacterial immune response is to blame for the pathogenesis of adult pulmonary TB in immunocompetent hosts. Direct and indirect evidence supporting this notion is presented in this manuscript. Protective immunity in TB is briefly described in the beginning, which is followed by the findings favoring the pathological role of hyperactive immune response in adult pulmonary TB. Finally, heterogeneity of TB and its role in complicating TB immunobiology has been discussed in short.

## Protective immune response against *Mtb*

The immune response to *Mtb* begins with inhalation of bacilli-laden droplets, produced by an active TB patient while coughing. Inside lungs, *Mtb* is recognized and phagocytosed by resident macrophages. These cells respond to *Mtb* infection by producing proinflammatory cytokines and chemokines, reactive nitrogen/oxygen species and antimicrobial peptides [2]. Monocytes extravasate into infected tissue, and under the influence of locally present cytokines and growth factors, mature into macrophages and dendritic cells (DCs) [3]. After migrating to the draining lymph nodes, infected and antigen-loaded DCs mount *Mtb*-specific CD4^+^ and CD8^+^ T cell responses.

CD4^+^ T lymphocytes are the key orchestrators of protective immunity against *Mtb* [2]. The critical role of these cells in protection against *Mtb* is evidenced by CD4^+^ T cell-lymphopenic HIV-infected people, who have 20–35 times higher risk of TB, compared with uninfected individuals [4]. CD4^+^ T cells act primarily by activating macrophages through soluble factors like IFN-gamma and TNF-alpha, and cell surface molecules such as CD40 [2]. Activated macrophages are capable of restricting *Mtb* growth and killing the intracellular bacilli in an inducible nitric oxide synthase (iNOS)-dependent manner. Activated macrophages have also been shown to enforce phagolysosomal fusion, which is otherwise inhibited by the pathogenic mycobacteria.

Proinflammatory cytokines TNF-alpha and IFN-gamma are the important mediators of protective immunity against *Mtb* [5]. TNF-alpha is produced mainly by macrophages, neutrophils, DCs and T cells. It activates macrophages and induces chemokine secretion by them, resulting in recruitment of other cells to the site of infection [6]. Further, apoptosis of *Mtb*-infected macrophages is enhanced in the presence of TNF-alpha, leading to the elimination of intracellular *Mtb* [7]. TNF-alpha has also been suggested to play a role in the granuloma formation and maintenance [8]. The importance of TNF-alpha in protection against *Mtb* is highlighted by reactivation of latent infection in people (such as rheumatoid arthritis patients), who have been treated with anti-TNF agents [9].

In *Mtb*-infected hosts, IFN-gamma is mainly produced by CD4^+^ T cells, although CD8^+^ T cells, γδT cells, NK cells and NKT cells also contribute towards it [10]. Production of IFN-gamma is largely dependent on IL-12, which is secreted by *Mtb*-infected DCs and macrophages. Mutations in IL-12/IFN-gamma axis predispose the affected individuals to progressive mycobacterial infection [11]. A partial loss of IFN-gamma receptor I (IFNGR1) enhances susceptibility to disseminated disease with BCG and environmental mycobacteria, whereas complete loss of IFNGR1 has been shown to result in earlier onset of disease symptoms [12]. Similarly, children with IL-12 receptor β1 (IL-12Rβ1) deficiency have been shown to develop the severe form of TB, frequently [13].

The coordinated response of innate and adaptive immune systems against *Mtb* results in the well-organized cellular fortress, which can effectively shield healthy lung tissue from *Mtb*. This fortress, referred to as granuloma, has infected macrophages, neutrophils, giant cells and epithelioid cells in its core, which is surrounded by CD4^+^ T cells, CD8^+^ T cells, B cells, and fibroblasts [14]. Granuloma provides a special framework, wherein infected macrophages remain sequestered from healthy tissue, while maintaining the close contact with effector T cells [15]. Most of the latently infected people with *Mtb* residing in their granulomas do not develop active disease and remain healthy lifelong, illustrating the protective role of granulomas against *Mtb*.

## Hyperactive immune response as an etiologic agent in adult pulmonary TB

Like any other thing in excess, the excessive immune response is not good for health and it holds true for the antimycobacterial immune response as well. Studies have shown that most of the adults pulmonary TB patients are not immunodeficient and are not susceptible to other infectious diseases in general. On the other hand, a number of evidence shows that a hyperactive anti-*Mtb* immune response, characterized by excessive CD4^+^ T cell activity and IFN-gamma levels, is to blame for the pathogenesis of adult pulmonary TB in immunocompetent hosts. This evidence is presented below.

### Lessons from tuberculin skin test

Tuberculin skin test (TST) is a widely used assay to diagnose exposure to *Mtb*. It involves the intradermal injection of purified protein derivative (PPD) and monitoring for a delayed-type hypersensitivity (DTH) reaction, which is seen as the local skin induration (tuberculin reaction) [16]. DTH is a cellular immune response, primarily involving CD4^+^ T cells and macrophages, against an antigen (or antigens) to which an individual is prior sensitized. The occurrence of an intense DTH reaction in adult pulmonary TB patients, as demonstrated by the large area of induration, shows that these individuals mount and maintain the higher levels of cellular immune responses to *Mtb* antigens [17].

Studies on the close contacts of TB patients have shown that those who initially exhibited a greater sensitivity to tuberculin carry the higher risk of developing active TB [18]. Accordingly, an intense tuberculin reaction is considered as more serious, and more likely indicate that a person is suffering from active disease or is about to develop it [19]. On the other hand, latently infected people exhibit a relatively lower sensitivity to *Mtb* antigens, indicating that a moderate anti-*Mtb* immune response is able to confer protection against active disease [20].

It should be noted that even though the intensity of tuberculin reaction correlates with the risk of pulmonary TB, lack of reactivity or anergy to *Mtb* antigens is not an indicative of resistance to the disease [21]. Anergy to *Mtb* antigens can be observed in patients suffering from HIV/AIDS or other forms of immunodeficiencies, but its underlying mechanisms in every subset of patients are not well-understood [22]. As anergy to *Mtb* antigens signifies the lack of T_H_1 type of antimycobacterial immune response, it can be associated with the unrestricted growth of the bacilli in any tissue/organ and the increased risk of mortality [21]. Nevertheless, the occurrence of intense tuberculin reaction in adult pulmonary TB patients demonstrates a strong association between heightened antimycobacterial immune response and the active disease.

### Immune profiling of latent and active TB patients

The antimycobacterial immune response has been characterized in latent and active TB patients with an aim to understand underlying mechanisms of TB pathogenesis. These studies have shown that heightened levels of IFN-gamma are most invariably observed in lung tissue, bronchoalveolar lavage (BAL) fluid, pleural effusion, and lymph nodes of active TB patients [5]. Interestingly, BAL fluid IFN-gamma levels of active TB patients directly correlate with disease severity and subside with successful chemotherapy [23]. Few studies have also demonstrated enhanced levels of IFN-gamma in blood-plasma of active TB patients, compared with healthy controls [24]. Similar to findings in human patients, increased IFN-gamma levels have also been observed in *M. bovis*-infected calves, which are natural hosts of these bacilli [25]. IFN-gamma secretion by *M. bovis*-specific T cells from these animals was also found to correlate with disease severity.

The above findings are further corroborated by transcriptional profiling of immune response in active TB patients. Microarray analysis of whole blood cells by Berry et al. has revealed an increased transcription of IFN-gamma- and type I IFN-inducible genes in active TB patients, compared with latently infected people and healthy controls [26]. Although, the occurrence of type I IFN-inducible transcriptomic signature in active TB patients was a surprising finding, the presence of IFN-gamma-inducible signature correlated with the heightened IFN-gamma levels in these people. Interestingly, as in the case of IFN-gamma levels in different tissues, IFN-inducible transcriptomic signature was also found to correlate with disease severity and subsided with successful chemotherapy. An interesting observation in these studies was that IFN-inducible transcriptomic signature was absent from most, but 10–20 % of latently infected people, who might be representing the transition state between latent and active TB [26]. Resolution of microarray data to cellular level showed that IFN-inducible genes in active TB patients were predominantly expressed in neutrophils, and to some extent in monocytes [26]. The presence of heightened IFN-gamma levels and its inducible transcriptomic signature in TB patients corroborated an association between hyperactive immune response and the active pulmonary disease.

### Course of Mtb infection in immunocompetent adults

*Mtb* infection in otherwise healthy immunocompetent adults follows a definite pattern. Initial exposure to *Mtb* in these people is followed by an asymptomatic phase of infection called latent TB (LTB) [2]. Although most immunocompetent adults with LTB would remain healthy, 5–10 % of them are destined to develop active disease in their lifetime. The risk of developing the active disease is maximum within 1–2 years post-infection and declines thereafter [18, 27]. There is almost always a time lag between *Mtb* exposure and active disease in immunocompetent adults, which probably represents the time period required for host antimycobacterial immune response to accumulating and reach pathological intensity.

A peculiar feature of adult pulmonary TB is that people who have been cured of active disease do not develop a resistance to subsequent infection. Instead, it has been observed that these people carry a significantly elevated risk of developing active disease with *Mtb* re-exposure [28]. This aspect of TB, although unexplained so far, supports the pathological role of immune response in the adult pulmonary disease. In fact, *Mtb*-specific lymphocytes have been found to persist as memory cells for an extended period of time after successful chemotherapy [24, 29]. Re-stimulation of these memory cells with *Mtb* antigens is bound to result in a rapid and fully blown antimycobacterial immune response. If the weakened immune response were responsible for the pathogenesis of adult pulmonary TB, activation of memory cells would have conferred protection against active disease. However, pulmonary TB in immunocompetent adults being a manifestation of the hyperactive antimycobacterial immune response, cured TB patients are doomed to suffer again.

### Immune reconstitution-induced TB (IR-TB) in HIV/AIDS patients

HIV/AIDS, which is characterized by reduced levels of CD4^+^ T cells, offers a unique opportunity to understand the role of immune system in TB pathogenesis. It allows us to examine the effect of a weakened as well as a heightened immune response on the outcome of mycobacterial infection. Three possible outcomes of *Mtb* co-infection in HIV/AIDS patients are: (i) progressive primary TB (ii) progressive latent TB and (iii) immune reconstitution-induced TB (IR-TB).

Given the central importance of CD4^+^ T cells in containing *Mtb* infection, the occurrence of a progressive primary TB in HIV/AIDS patients is conceivable. Progressive latent TB in HIV/AIDS patients is similar to progressive primary TB except that *Mtb* infection, in this case, is established before HIV-mediated immunosuppression. The unrestricted growth of bacilli in affected tissue(s) is responsible for disease symptoms in both progressive primary and progressive latent TB.

Immune reconstitution-induced TB (IR-TB), also known as tuberculosis-associated immune reconstitution inflammatory syndrome (TB-IRIS), is a special case of the disease observed in HIV- and *Mtb*-coinfected people treated with anti-retroviral therapy (ART) [30]. It occurs in diverse manifestations, including abdominal lymphadenopathy, pulmonary or pericardial effusions, brain tuberculomas, and skeletal lesions [31]. IR-TB affects up to 45 % the coinfected people and poses a major challenge in the clinical management of HIV infection in them [32]. Interestingly, IR-TB provides unequivocal evidence favoring the pathological role of hyperactive immune response during *Mtb* infection.

Administration of ART in HIV-infected people reduces viral load, leading to a rapid increase in CD4^+^ T cell count. The presence of *Mtb* antigens at this stage results in the selective expansion of *Mtb*-specific CD4^+^ T cells [33, 34]. Studies have shown that both terminally-differentiated and effector memory CD4^+^ T cells with specificity to *Mtb* antigens are expanded in the co-infected people receiving ART [35, 36]. Consistent with these findings, conversion from a ‘negative’ TST status to a ‘strongly positive’ one has been observed in ART-treated HIV- and *Mtb*-coinfected people. Most importantly, it has been demonstrated that the co-infected people who develop IR-TB possess higher levels of circulating *Mtb*-specific CD4^+^ T cells, compared with those who did not experience this condition [34, 37, 38]. These immunological features in the affected individuals clearly show that IR-TB is driven by a hyperactive CD4^+^ T cell response against *Mtb* antigens.

Mechanistic insights into IR-TB have been gained with a recently developed mouse model [39]. In this model, Barber et al. mimicked human IR-TB by adoptively transferring a small number of naive CD4^+^ T cells into *M. avium*-infected, T cell-deficient (TCRαKO) mice. It was observed that donor CD4^+^ T cells underwent a rapid expansion in *M. avium*-infected TCRαKO mice and led to the failure of lung function, wasting and eventual death. The authors further demonstrated that the ability to cause lung pathology was lost in IFN-gamma-deficient CD4^+^ T cells [39]. Thus, studies in both IR-TB patients and the animal model demonstrate the pathological role of hyperactive, IFN-gamma-producing CD4^+^ T cells during *Mtb* infection.

### Curative effects of vitamin D and anti-inflammatory drugs against TB

Vitamin D is known to possess curative effects against TB since a long. Low serum level of vitamin D is an important risk factor for TB, and polymorphisms in both vitamin D receptor (VDR) and vitamin D-binding protein are associated with increased TB susceptibility [40, 41]. When evaluated as an adjunct to standard TB therapy, vitamin D has resulted in accelerated sputum conversion and lung healing [42]. Usefulness of vitamin D against TB, also observed in a host of other studies, has been attributed to its anti-inflammatory properties [43].

Vitamin D dampens both innate and adaptive immune responses [43, 44]. By promoting IκB expression, it prevents nuclear translocation of NF-κB and expression of genes involved in the inflammatory process. Vitamin D has also been shown to inhibit proliferation of VDR-expressing T cells and induction of T_H_1 type of immune responses [45]. Negative regulatory effects of vitamin D on T_H_1 type of immune response can be partly attributed to IL-10 [46, 47]. Interestingly, metabolites of vitamin D have been shown to increase the number and functions of CD4^+^ FoxP3^+^ T regulatory cells (Tregs), which are the important source of IL-10 [48, 49]. These effects of vitamin D on Tregs are highly relevant, as the latency of *Mtb* infection has been shown to be associated with increased frequency of Tregs in lung tissue and bronchoalveolar lavage [50, 51].

Similar to vitamin D, steroid and non-steroid anti-inflammatory drugs are known to possess ameliorating properties against TB. Corticosteroids are frequently used as an adjunct to standard therapy for treatment of various forms of TB and its associated complications, including IR-TB [52, 53]. They have been shown to improve TB prognosis and to reduce TB-associated mortality [54]. Similarly, administration of ibuprofen, a non-steroidal anti-inflammatory drug (NSAID), has been shown to result in significantly reduced bacillary load and increased survival of C3HeB/Fej mice, which mimics the granulomatous response of human TB [55]. Administration of diclofenac has also resulted in decreased number of colony-forming units (CFUs) in spleen and liver of *Mtb*-infected animals [56].

The anti-inflammatory properties of steroids and NSAIDS have been implicated in their beneficial effects against TB. NSAIDs inhibit COX-1 and COX-2, and reduce biosynthesis of prostaglandins, which are involved in the cardinal signs of inflammation- redness, swelling and pain [57]. Interestingly, higher levels of prostaglandin E_2_ have been noted in sera of active TB patients, compared with healthy controls [58]. Corticosteroids suppress proinflammatory genes mainly by histone deacetylase-2 (HDAC2)-mediated reversal of histone acetylation at activated transcription complex [59]. Suppression of proinflammatory cytokines has been shown to be responsible for beneficial effects of prednisone against IR-TB [60].

The beneficial of vitamin D and other anti-inflammatory agents against TB shows that controlling the host immune response during *Mtb* infection results in the increased resistance to active disease or in its better prognosis. Therefore, the pathological role of hyperactive immune response in adult pulmonary TB in immunocompetent hosts is corroborated by the curative effects of vitamin D and other anti-inflammatory agents against this disease.

## Heterogeneity of tuberculosis

Our understanding of TB immunopathology has been complicated largely by the heterogeneity of this disease [5]. Latent TB and active TB are two major outcomes of *Mtb* infection in humans. Active TB further manifests itself in different forms including primary TB in young children, disseminated TB in AIDS patients, reactivated TB in immunocompetent adults, and IR-TB in HIV-infected people receiving ART [5]. Heterogeneity of active TB is also observed in affected organs. TB in immunocompetent adults primarily affects lung tissue, whereas in young children and immunocompromised people, it frequently occurs in extrapulmonary form [61]. Different presentations of TB are the outcomes of the specific interaction between host immune response and *Mtb*.

The immune response against *Mtb* could be hypoactive, balanced or hyperactive. Upon initial exposure to *Mtb*, immunocompetent adults mount a balanced antimycobacterial immune response, resulting in containment of bacilli in lung granulomas and protection against active disease [62]. Depending on host immunoregulatory factors and/or exposure to external agents, the antimycobacterial response could diminish or aggravate. In latently infected people destined to develop active TB, antimycobacterial immunity intensifies with time and after crossing a threshold, leads to the immunopathology. As discussed above, similar aggravation of anti-*Mtb* immune response also takes place in AIDS patients receiving ART.

The primary, disseminated and severe nature of TB in young children demonstrates the incompetence of their immune system to contain the bacilli. Interestingly, it has been observed that younger is a child, higher is the risk of TB. This pattern of disease occurrence in children suggests that resistance to TB develops with age (till adolescence) [63]. Although the exact nature of immune-incompetence in young children remains unclear, the protective efficacy of BCG against childhood TB indicates the lack of effective T cell-mediated immunity in them [64]. A role of DCs has also been implicated in the inability of young children to mount an effective T cell response against *Mtb* [64]. Similar to TB in young children, various forms of immune-deficiencies such as inherited IFN-gamma deficiency and HIV-mediated immunosuppression also result in the extrapulmonary and/or disseminated TB.

It is noteworthy that outcome of the interaction between immune system and *Mtb* is shaped by multiple host factors, including age, diet, body mass index, genetic makeup, other chronic diseases or co-infections and exposure to environmental mycobacteria [65]. Multi-factorial influence on the outcome of *Mtb* infection may sometimes defy the generalizations in TB immunopathology. Owing to the unrestricted growth of the bacilli in immunodeficient hosts, *Mtb* infection in a certain proportion of these people can result in the pulmonary disease [66]. Alternatively, a proportion of extrapulmonary TB cases could be attributed to hyperactive antimycobacterial immune response.

## Conclusion and future perspectives

The above evidence establishes that a hyperactive antimycobacterial immune response, characterized by heightened CD4^+^ T cell activity and IFN-gamma levels, is to blame for the pathogenesis of adult pulmonary TB in immunocompetent hosts. This evidence is provided by both observational and experimental studies on human subjects and contradicts the commonly held view that weakening of immune system leads to reactivation of latent *Mtb* infection into the active disease. Although the risk of TB is increased with the weakening of immune system, TB affecting the immunocompromised hosts is different from that occurring in immunocompetent adults. In immunocompromised hosts and young children, uncontrolled growth of the bacilli leads to the disease development, whereas in immunocompetent adults, immune system-mediated damage to lung tissue is to blame for the TB pathology. Different mechanisms of disease development are responsible for the heterogeneity of TB, which is being appreciated by an increasing number of researchers.

The view presented in this manuscript has a direct bearing on the course of TB vaccine development programs. Owing to the different mechanisms of pathogenesis, different vaccination approaches are required against different forms of TB. The antimycobacterial immunity needs to be boosted in young children and tamed in the immunocompetent adults. Unfortunately, the vaccinologists had been trying to boost the anti-*Mtb* immune response, without taking into consideration the heterogeneity of TB and its mechanisms of pathogenesis. Although such vaccines could be effective in the young children, as in the case of BCG, they are destined to fail in the immunocompetent adults.

Conclusively, if we wish to save another 100 years, billions of dollars, and most importantly, the millions of lives, it is high time to change our perspective of TB immunopathology. It is time to appreciate that TB is a heterogeneous disease and that different vaccination approaches are required to control its different forms. For TB continues to be a major public health threat with its drug-resistant form spreading at an alarmingly high rate, the onus of controlling the crisis lies with the immunologists.

## Abbreviations

TB: tuberculosis
*Mtb*: *Mycobacterium tuberculosis*
BCG: Bacillus Calmette–Guérin
DTH: delayed-type hypersensitivity
LTB: latent tuberculosis
IR-TB: immune reconstitution-induced tuberculosis
NSAIDs: non-steroidal anti-inflammatory drugs
ART: antiretroviral therapy
